# The *in Vitro* and *in Vivo* Degradation of Cross-Linked Poly(trimethylene carbonate)-Based Networks

**DOI:** 10.3390/polym8040151

**Published:** 2016-04-19

**Authors:** Liqun Yang, Jianxin Li, Miao Li, Zhongwei Gu

**Affiliations:** 1 Key Laboratory of Reproductive Health and Medical Genetics, National Health and Family Planning Commission, Shenyang 110031, China; yanglq@lnszjk.com.cn (L.Y.); jxinl@vip.sina.com (J.L.); limiao_lnszjk@163.com (M.L.); 2 Key Laboratory of Reproductive Health, Liaoning Research Institute of Family Planning, Shenyang 110031, China; 3 National Engineering Research Center for Biomaterials, Sichuan University, Chengdu 610064, China

**Keywords:** cross-linked networks, poly(trimethylene carbonate), *in vitro* degradation, *in vivo* degradation, form-stability

## Abstract

The degradation of the poly(trimethylene carbonate) (PTMC) and poly(trimethylene carbonate-*co*-ε-caprolactone) (P(TMC-*co*-CL)) networks cross-linked by 0.01 and 0.02 mol % 2,2′-bis(trimethylene carbonate-5-yl)-butylether (BTB) was carried out in the conditions of hydrolysis and enzymes *in vitro* and subcutaneous implantation *in vivo*. The results showed that the cross-linked PTMC networks exhibited much faster degradation in enzymatic conditions *in vitro* and *in vivo versus* in a hydrolysis case due to the catalyst effect of enzymes; the weight loss and physical properties of the degraded networks were dependent on the BTB amount. The morphology observation in lipase and *in vivo* illustrated that enzymes played an important role in the surface erosion of cross-linked PTMC. The hydrolytic degradation rate of the cross-linked P(TMC-*co*-CL) networks increased with increasing ε-caprolactone (CL) content in composition due to the preferential cleavage of ester bonds. Cross-linking is an effective strategy to lower the degradation rate and enhance the form-stability of PTMC-based materials.

## 1. Introduction

Biodegradable polymers have been extensively investigated for biomedical and pharmaceutical applications including implanted medical devices [1,2,3,4,5,6], drug delivery systems [7,8,9,10,11,12,13], nerve guides [14,15,16] and temporary three-dimensional (3D) scaffolds in tissue engineering [17,18,19,20,21,22]. Poly(trimethylene carbonate) (PTMC) is one of the most important biodegradable polymers due to its favorable characteristics, such as excellent degradability and biocompatibility [23,24,25]. In previous work, it has been demonstrated that PTMC was hardly degraded in aqueous solutions [23,26], whereas its degradation was rapid *in vivo* via surface erosion mechanism [23,25]. The degradation products of PTMC were not acidic, which was much better than polyesters at avoiding the inflammation [27,28,29,30] led by acidic degradation products. PTMC was degraded when incubated in lipase solutions (*from Thermomyces Lanuginosus*) [23]. Once degraded, PTMC loses it shape due to the lack of structural stability [31], which is undesirable to limit the applications in medical implants. Hence, the enhancement of form-stability was considered as one of effective strategies to improve the *in vivo* usability of PTMC.

PTMC-based biodegradable cross-linked networks (BCNs) have been reported to enhance the mechanical and degradation properties of PTMC. The γ irradiation cross-linked PTMC network [32] showed that the cross-linking degree of networks increased with increasing the radiation dose and the mechanical properties could be strengthened. Erhan Bat *et al.* [33,34] cross-linked melt pressed (co)polymer films of trimethylene carbonate (TMC) and CL via γ irradiation in vacuum and investigated the *in vitro* and *in vivo* degradation of the networks. The results revealed that the degradation of networks was in accordance with a surface erosion mechanism, and the erosion rates could be controlled by the TMC/CL ratio and the irradiation dose. Furthermore, histological evaluation indicated that the networks initiated a mild tissue response when implanted intramuscularly in rats [34]. Similar to the gamma irradiated PTMC networks, BCNs based on UV-photocrosslinked star copolymers of TMC and CL also have been demonstrated to be well tolerated by the host tissue [35]. Therefore, PTMC-based BCNs possessed excellent tensile strength, flexibility and controllable degradation rates, and have potential applications in a broad range of medical fields [36,37,38,39,40].

Recently, we fabricated PTMC-based BCNs using a new reaction methodology of cross-linking agent other than γ irradiation to avoid the chain scission during the cross-linking [41]. In addition, 2,2′-bis(trimethylene carbonate-5-yl)-butylether (BTB) synthesized from di(trimethylene propane) and ethyl chloroformate was used as cross-linker in the (co)polymerization of TMC and ε-caprolactone (CL) in the presence of stannous octanoate (Sn(Oct)_2_). The effects of cross-linker amount and CL content on the performance of the resulted elastomers were investigated. A series of structurally stable networks with desired mechanical and thermal properties were received. In this paper, both *in vitro* and *in vivo* degradation of the cross-linked PTMC-based BCNs were studied. The *in vitro* degradation was performed in lipase solutions (from *Thermomyces lanuginosus*, ≥100,000 U/g) and phosphate buffered saline (pH = 7.4) at 37 °C. The *in vivo* degradation was carried out in subcutaneous implantation. The form-stability and tissue responses of the implanted networks were studied as well.

## 2. Materials and Methods

### 2.1. Materials

TMC was purchased from Daigang Biomaterial Co., Ltd. (Jinan, Shandong, China), recrystallized twice in ethyl acetate and vacuumed dried at 37 °C for 24 h before polymerization. ε-caprolactone (99%) was purchased from Sigma-Aldrich (St Louis, MO, USA), freshly distilled over CaH_2_ under reduced pressure before use. Sn(Oct)_2_ (95%) was purchased from Sigma-Aldrich and used as received. All other solvents and reagents were analytical grades and purified by standard methods.

### 2.2. Methods

^1^H–NMR spectra were recorded on a Bruker ARX 300 (Bruker, Zurich, Swiss) using CDCl_3_ as solvent with tetramethylsilane (TMS) as an internal standard. The molecular weight and distribution (*M*_n_ and PDI) of the sol content were determined by GPC (Waters, Milford, MA, USA) with a Waters Model 1515 isocratic high-performance liquid chromatography (HPLC) pump, a Waters Model 2414 differential refractive index detector and a Waters Styragel HT4 chromatographic column. THF was used as eluent with a flow rate of 1 mL/min at 35 °C. The molecular weight and molecular weight distributions were calculated using polystyrene as standard. Glass transition temperature of the networks was determined with a Netzsch DSC 200 F3 (Netzsch, Selb, Germany) equipped with a liquid nitrogen cooling system. The measured temperature range was between −100 and 100 °C and the heating rate was 10 °C/min under nitrogen atmosphere. The thermal stability of the polymers under nitrogen atmosphere was carried out by a Netzsch TGA 209 F3 (Netzsch, Selb, Germany) at a temperature ranging from room temperature to 550 °C and the heating rate was 10 °C/min. Tensile testing was performed on the obtained networks measuring approximately 40 × 3 × 2 mm^3^ using an Instron 1121 universal testing machine (Instron, Grove, PA, USA) with a crosshead speed of 50 mm/min in accordance with GB/T 1040.1-2006. The tests were done on triplicate samples, and the results were presented as an average for tested samples. The scanning electron microscopy (SEM) micrographs were obtained from a XL30ESEM–FEG microscope (FEI-Philips, Eindhoven, The Netherlands). The sample surfaces were sputter coated with Au to avoid charging.

### 2.3. Preparation of Network Films

**Figure 1:**
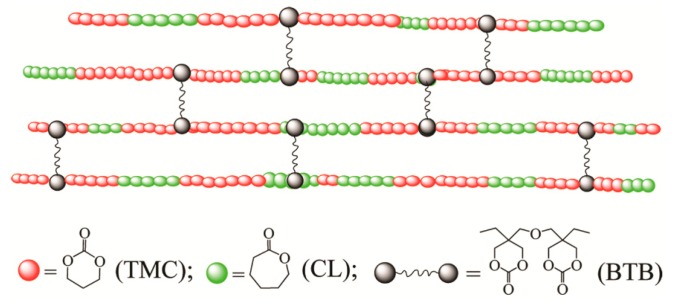
Schematic representation of the formation of the P(TMC-*co*-CL) networks cross-linked by BTB.

A mixture of monomers (TMC and/or CL) and BTB (prepared and characterized as described previously [41]) was charged into pre-silanized flat-bottomed ampoules with a diameter of 60 mm under nitrogen atmosphere, 2 × 10^−4^ mol of Sn(Oct)_2_ per mole of monomers in anhydrous toluene was added. The toluene was removed by vacuum evaporation. The ampoules were purged three times with dry nitrogen and heat-sealed under vacuum. The ampoules were conditioned in an oil-bath pre-heated at the polymerization temperature and shaken vigorously to mix the mixture homogeneously. The ampoules were put in the oil bath for polymerization. All copolymerizations were carried out at 130 ± 2 °C for 24 h. After the reaction, the ampoules were quenched to room temperature, and the films were discharged and easily removed from the ampoules. Figure 1 shows the schematic representation of the formation of the cross-linked P(TMC-*co*-CL) networks.

To determine the gel percentage of the network films, a piece of sample was weighed and kept in a sealed flask containing chloroform for 1 week and the solvent was refreshed twice a week to remove the sol fraction completely. The swollen gels were taken out and dried to constant weight at 37 °C in vacuum. The gel and the sol fractions were calculated according to Equations (1) and (2), respectively:

(1) $$\text{gel percentage}(\%)=\frac{w_{d}}{w_{i}}\times 100,$$

(2) $$\text{sol percentage}(\%)=\left(1-\frac{w_{d}}{w_{i}}\right)\times 100,$$

where *w*_d_ is the mass of dried swollen samples and *w*_i_ is the mass of the specimens before swelling. The measurement for gel and the sol fractions was done in triplicate for each network sample.

### 2.4. In Vitro Enzymatic Degradation

Circular specimens with a diameter of 10 mm were punched out of the films, weighed and put in lipase solutions (from *Thermomyces lanuginosus*, ≥100,000 U/g), the media were refreshed twice a week. The degradation experiments were performed in triplicate at 37 °C with gentle shaking. At regular time intervals, the polymer specimens were taken out from the degradation media, blotted with a tissue and weighed. The specimens were washed with deionized water and then vacuum-dried at 37 °C till constant weight. The water uptake and mass loss were calculated according to the following equations:

(3) $$\text{Water uptake}(\%)=\frac{w_{w}-w_{d}}{w_{d}}\times 100,$$

(4) $$\text{Mass loss}(\%)=\frac{w_{i}-w_{d}}{w_{i}}\times 100,$$

where *w*_i_, *w*_w_, and *w*_d_ represent the initial weight, wet weight and dry weight of the samples, respectively.

### 2.5. In Vitro Hydrolytic Degradation

Strip shaped specimens with dimensions of approximately 40 × 3 × 2 mm^3^ were cut from the films for hydrolytic degradation study. The samples were immersed into vials containing 20 mL of pH 7.4 phosphate buffered saline (PBS), which was replaced once a week and the vials were placed in an incubator at 37 °C. At time points of 10, 15, 20, 25 and 30 weeks, the samples were removed from PBS and washed with deionized water. After wiping, the specimens were weighed and dried at 37 °C in vacuum till constant weight. Mass loss and water uptake were then determined.

### 2.6. In Vivo Degradation

Before subcutaneous implantation, strip shaped specimens with dimensions of approximately 40 × 3 × 2 mm^3^ were sterilized by soaking in 75% ethanol for 3 h, rinsed with deionized water and air-dried in a sterile environment. Animal experiments were performed according to the Regulations of Experimental Animal Administration issued by The people’s Government of Liaoning province (Decree No. 143 of 1 October 2002). Adult male Wistar rats with body weight of 200 g were used. Four specimens of one sample were implanted in the back of one rat for each time point.

After implanted for 2, 4, 8 10, 12 and 14 weeks, the rats were sacrificed and shaved, and the surrounding tissues of implants were excised. Three implants of one polymer in each study point were pulled out from the fibrous tissue capsules gently with tweezers and one with surrounding tissue was used for histological examination. Silastic tubes were used as positive controls.

The explants were fixed in neutral buffered formalin for 24 h. After being rinsed in distilled water and dehydrated in graded alcohol solutions, the explants were embedded in paraffin wax. Histological sections (5 μm) were routinely stained with hematoxylin eosin (HE). The histology was independently evaluated by two persons.

## 3. Results and Discussion

### 3.1. Synthesis of Cross-Linked PTMC Networks

To enhance the form-stability and control the degradation rate of PTMC for implant applications, PTMC-based cross-linked networks were prepared using BTB as cross-linker (Table 1). After copolymerization, the products were isolated as transparent solids and insoluble partially in chloroform. To determine the gel and sol fractions, the products were thoroughly washed and extracted with chloroform and there was weak gel left, which was easily broken when being picked up with tweezers due to low gel percentage. The gel percentage of the products was listed in Table 1. Obviously, the TMC content influenced the gel percentage greatly, and higher TMC content resulted in higher gel percentage of the films, similar to the observation in our previous work [41]. This was attributed to the fact that BTB copolymerizes better with TMC because it has a similar reactivity to TMC monomer. Moreover, the gel percentage of the networks increased with increasing the BTB amount in feeding dose. After extraction, the soluble parts were collected by evaporation under reduced pressure and analysis by ^1^H–NMR. The results showed that there were no proton signals of BTB in the ^1^H–NMR spectrum, which implied the successful incorporation of BTB to the networks. The molecular weight of the sol fractions was also given in Table 1. The sol fractions had higher *M*_n_ and narrower PDI than that of sol fractions extracted from the networks cross-linked by gamma irradiation [33]. This indicated that the BTB as cross-linking agent could avoid chain scission during cross-linking.

The thermal and mechanical properties of the network films were shown in Table 1. The cross-linked P(TMC-*co*-CL) networks containing 100, 75 and 50 mol % TMC were rubbery under physiological temperature and the *T*gs ranged from −16.2 to −44.1 °C. The *T*gs of cross-linked PTMC networks were slightly higher than that of non-crosslinked one due to the restricted motion of chain segments caused by cross-linking. A similar observation was found for the cross-linked P(TMC-*co*-CL) networks as compared to the linear P(TMC-*co*-CL) [42]. Furthermore, the *T*gs of cross-linked P(TMC-*co*-CL) networks decreased with increasing CL content, which was attributed to the low glass transition temperature of PCL (around −60 °C).

The obtained networks were flexible with elastic modulus ranging between 1.83 and 3.98 MPa. The elastic modulus and the tensile strengths of the networks increased with the increase of cross-linker amount while decreasing with the increase of CL content. It was due to the higher activity of the BTB, which was preferable to react with TMC monomer, thus resulting in less cross-linking points and low gel percentage in the network when CL content increased. The thermal and mechanical properties indicated that the PTMC-based cross-linked networks were elastic and flexible biomaterials potentially for subcutaneous implants.

### 3.2. In Vitro Enzymatic Degradation

**Figure 2:**
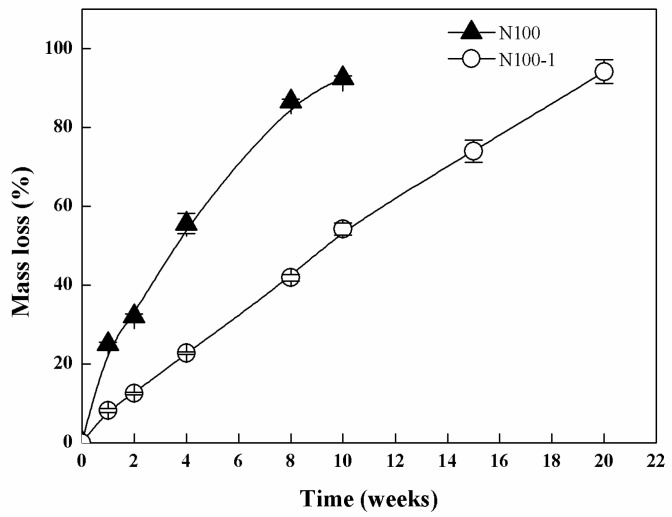
Mass loss of N100 and N100-1 specimens conditioned in lipase solutions at 37 °C for different time periods.

Lipase (*from Thermomyces lanuginosus*, ≥100,000 U/g) was used as a model enzyme to investigate the enzymatic degradation of the cross-linked PTMC. Figure 2 showed the mass loss of cross-linked N100-1 and non-crosslinked N100 specimens in lipase solutions.

As expected, N100-1 presented a slower mass loss than N100. After 10 weeks, the mass loss of N100-1 was 54.23% ± 1.56% and that of N100 was 92.44% ± 0.62%, and the N100 sample was almost eroded completely within 10 weeks. This observation showed that the cross-linked PTMC networks were more insensitive and resistant to lipase degradation. Cross-linking showed significant influence on the degradation behavior and could greatly lower the degradation rate of PTMC.

**Figure 3:**
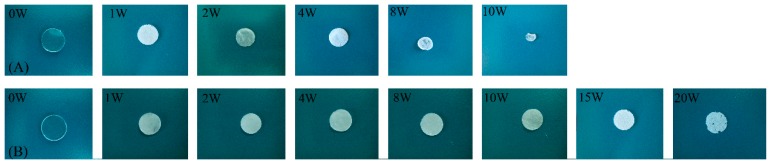
Shape of N100 (**A**) and N100-1 specimens (**B**) at different times of enzymatic degradation in lipase solutions. The initial diameter of the specimens was 10 mm.

**Figure 4:**
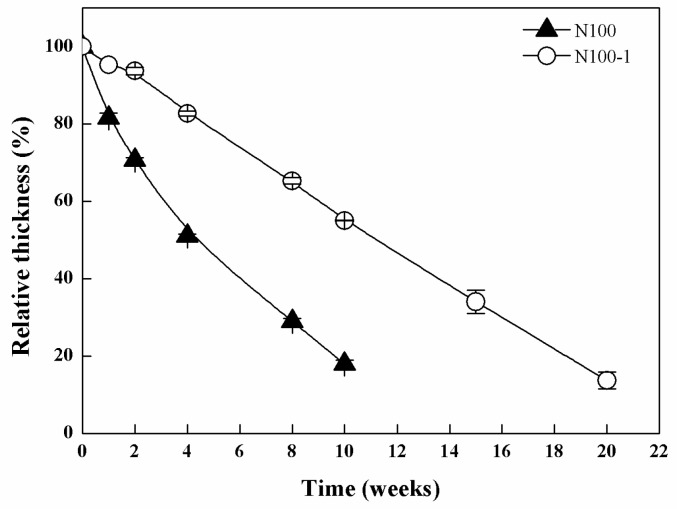
Relative thickness of N100 and N100-1 conditioned in lipase solutions at 37 °C for different times.

The macroscopic morphologies of the cross-linked PTMC network specimens degraded in enzymatic conditions were observed. As displayed in Figure 3A, the shape of N100 specimens was not circular anymore, but highly irregular after being degraded in lipase solutions for eight weeks. N100-1 exhibited better form-stability during incubating in the lipase solution at 37 °C for 15 weeks. The shape was not changed appreciably, and the diameter remained more or less constant (Figure 3B), while the thickness of the discs decreased with degradation time (Figure 4). At 20 weeks, the specimens of N100-1 were thin with serrate margins and visible pores on the surface. The change in the appearance of the N100 and N100-1 revealed that the cross-linked PTMC networks had a better form-stability than the non-crosslinked ones.

**Figure 5:**
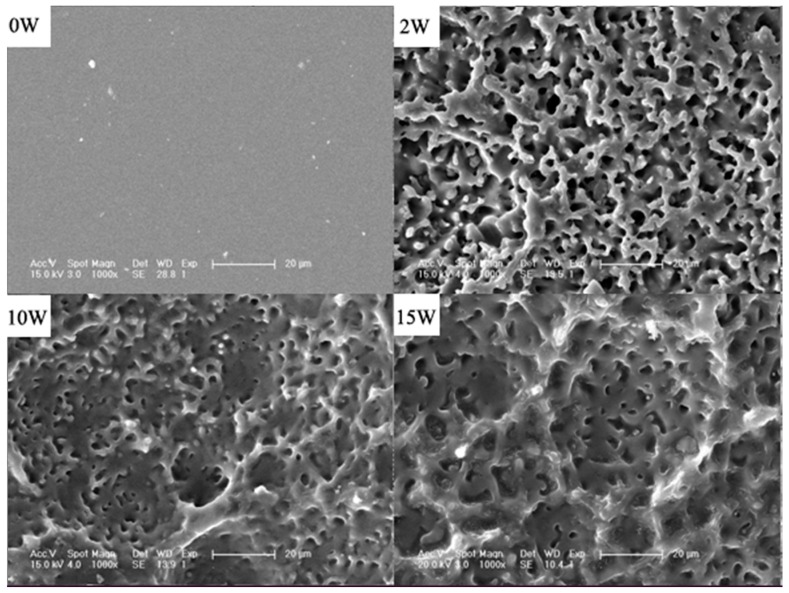
SEM micrographs of N100-1 before and after 2, 10 and 15 week enzymatic degradation. The scale bar was 20 μm.

SEM measurements were performed to examine the changes in surface morphology of the PTMC-based networks before and after degradation. The original samples of the cross-linked PTMC networks (N100-1) exhibited a smooth surface before enzymatic degradation. However, a highly porous structure was detected after 2, 10 and 15 weeks of degradation, and the size and deepness of the pits observed on the surface increased with the incubation time, as illustrated in Figure 5. The results were similar to the findings previously reported for PTMC [43], demonstrating that the cross-linked PTMC was degraded in lipase solutions via a surface erosion process.

**Figure 6:**
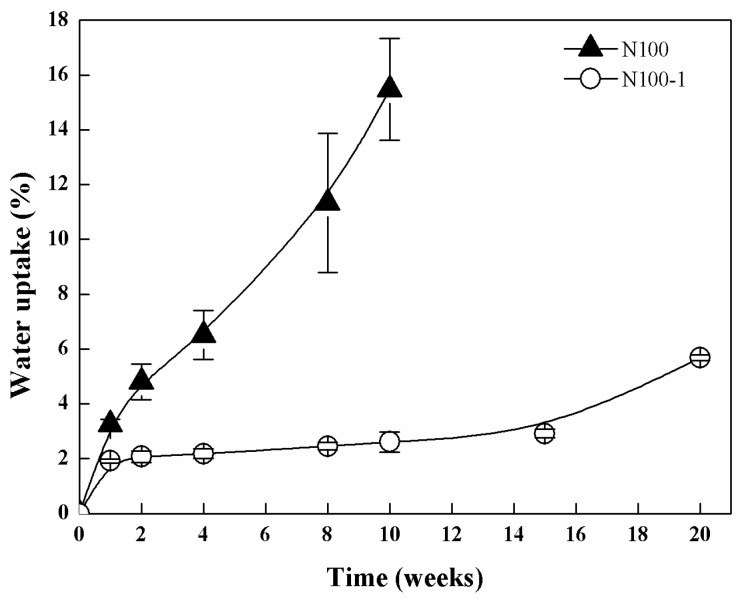
Water uptake of N100 and N100-1 specimens conditioned in lipase solutions at 37 °C for different time periods.

Figure 6 illustrated the water uptake curves during enzymatic degradation of the networks. N100 presented a gradual increase in water uptake; the water uptake was 15.47% ± 1.86% after 10 weeks and that of N100-1 was approximately 5.68% ± 0.11% after 20 weeks. The lower water uptake of cross-linked PTMC was in virtue of the fact that the cross-linked PTMC had a significantly slower degradation rate with less porous pits and holes on the surface (Figure 2), where the diffusivity of solvent molecules was less.

**Figure 7:**
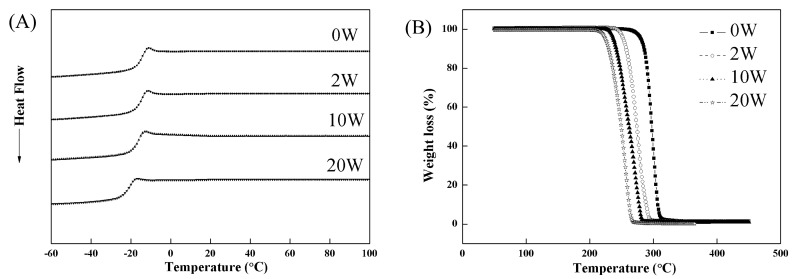
DSC (**A**) and TGA (**B**) curves of N100-1 conditioned in lipase solutions at 37 °C for different times.

The thermal properties of the cross-linked PTMC network (N100-1) *in vitro* enzymatic degradation were evaluated by DSC and TGA, and the representative curves were shown in Figure 7.

**Figure 8:**
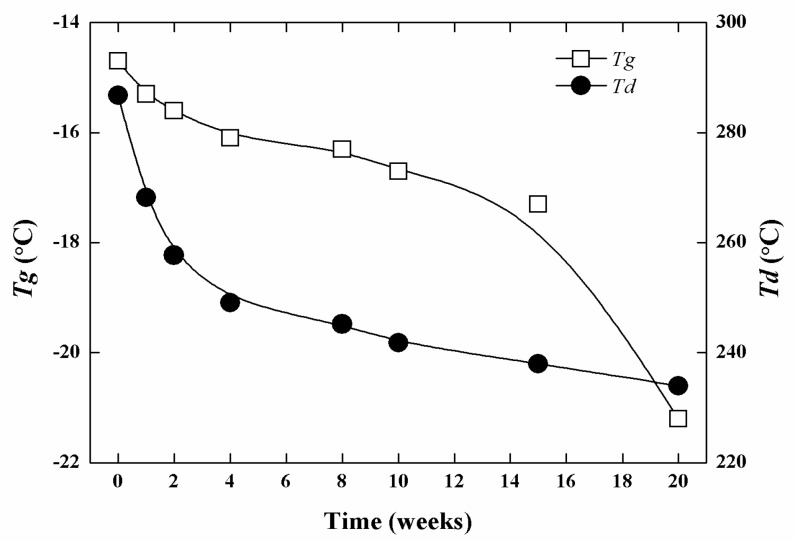
Change in thermal properties of N100-1 during *in vitro* enzymatic degradation in lipase solutions at 37 °C.

Figure 8 shows the changes in *T*g and *T*d of N100-1 during *in vitro* enzymatic degradation. Both the *T*g and *T*d decreased with increasing degradation time, and they were −21.2 and 234.0 °C after degradation for 20 weeks. This result indicated that the enzymatic degradation could destruct the structure and decrease the thermal stability of the cross-linked PTMC significantly.

The samples conditioned in the enzymolysis cases were too short and weak to be clamped for tensile test, hence their mechanical properties were not be measured.

### 3.3. In Vitro Hydrolytic Degradation

**Figure 9:**
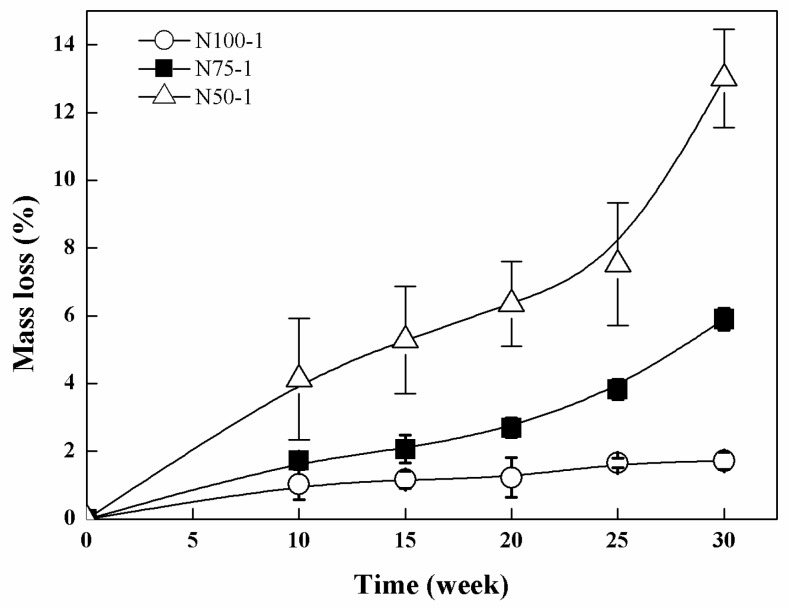
Mass loss of the cross-linked P(TMC-*co*-CL) networks during hydrolytic degradation.

The hydrolytic degradation of the PTMC-based networks was performed in pH 7.4 PBS at 37 °C. The cross-linked PTMC network N100-1 degraded extremely slowly due to the hydrophobic characteristics of PTMC [26], and the mass loss was only 1.71% ± 0.26% after 30 weeks of degradation. In contrast, the cross-linked P(TMC-*co*-CL) networks were hydrolytically degradable and the mass loss rapidly increased to 5.90% ± 0.32% for N75-1 and 13.01% ± 1.45% for N50-1 at week 30, as shown in Figure 9. Apparently, the cross-linked P(TMC-*co*-CL) networks degraded much faster than the cross-linked PTMC networks, the degradation rates of copolymer networks increased with the increase of the CL content due to the hydrolysis of ester bonds [27]. Furthermore, the mass loss of cross-linked PTMC networks in hydrolytic degradation was less than that in enzymatic degradation, implying the significant contribution of enzyme to the degradation of cross-linked PTMC networks.

**Figure 10:**
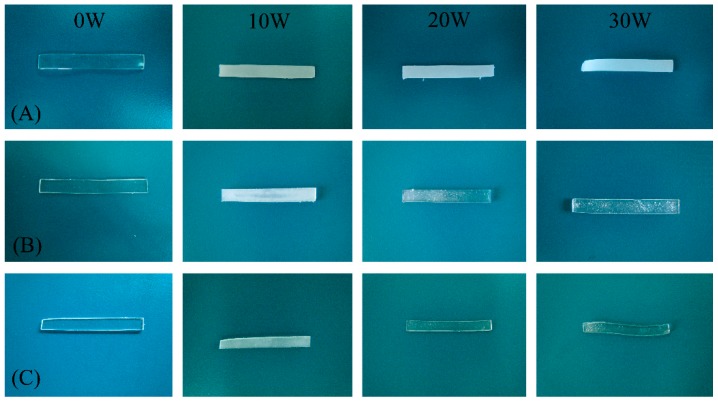
Macroscopic observation of the cross-linked P(TMC-*co*-CL) networks during hydrolytic degradation: (**A**) N100-1; (**B**) N75-1; and (**C**) N50-1.

The cross-linked P(TMC-*co*-CL) networks exhibited wonderful form-stability and no significant deformation was observed during the hydrolytic degradation process with the exception of N50-1 that became bended at 12 weeks (Figure 10). The slight deformation of N50-1 might be attributed to the lowest gel percentages and the fastest degradation rat. In our previous works, we reported that the non-crosslinked P(TMC-*co*-CL) copolymers presented poor form-stability and unexpectedly began changing their shape from a cylinder to an oblate sphere after 10 weeks *in vitro* hydrolytic degradation. The results indicated that cross-linking is an effective strategy to enhance the form-stability of the linear P(TMC-*co*-CL) copolymers.

**Figure 11:**
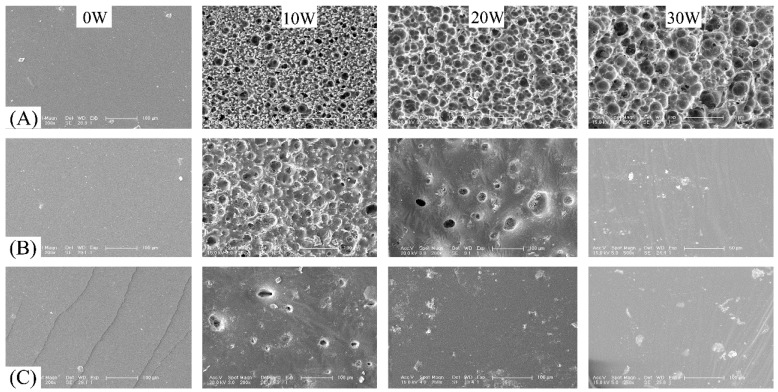
SEM micrographs of the cross-linked P(TMC-*co*-CL) networks before and after the *in vitro* hydrolytic degradation: (**A**) N100-1; (**B**) N75-1; (**C**) N50-1. The scale bar was 100 μm for all images with exception of 50 μm for N75-1 at week 30.

Similar to the enzymolysis cases, the surface of the cross-linked P(TMC-*co*-CL) networks before *in vitro* hydrolytic degradation was smooth, and it became rough and a number of pits were also visible on the surface after 10 weeks of degradation. For N100-1, the pits grew in size with degradation time (Figure 11A), indicating the further degradation. However, the pits size observed on the surface of N75-1 and N50-1 seemed to be smaller with degradation, and the surface became relative smooth (Figure 11B,C), although the mass loss increased significantly as shown in Figure 9. It was attributed to the viscous flow caused by the plasticizing effect of the low molecular weight degradation products leaching out of the degrading samples, which led to the structure collapse and formed the smooth surface.

**Figure 12:**
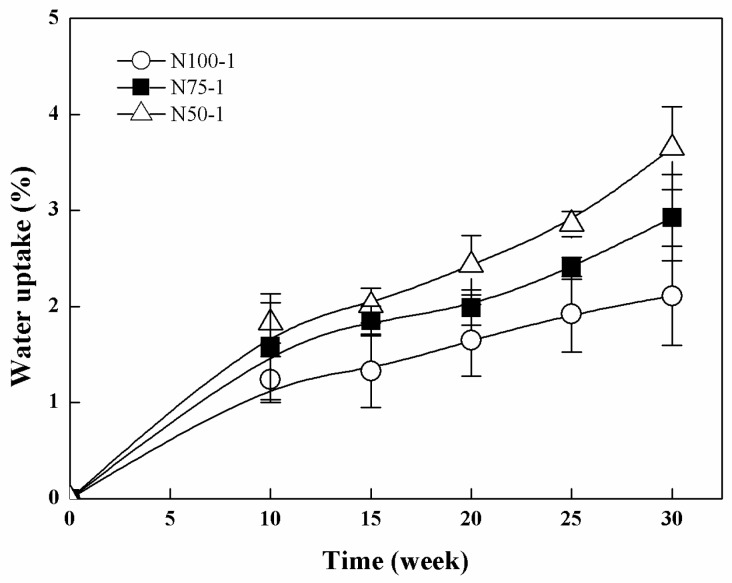
Water uptake s of the cross-linked P(TMC-*co*-CL) networks during hydrolytic degradation.

Figure 12 presents the water uptake profiles of the cross-linked P(TMC-*co*-CL) networks during hydrolytic degradation. N100-1 was very hydrophobic and its water uptake was 2.11% ± 0.52% after 30 weeks. N75-1 and N50-1 presented slightly higher water uptakes with 2.93% ± 0.45% and 3.65% ± 0.43%, respectively. The higher water uptake was due to the degradation of ester bonds in the cross-linked copolymers.

**Figure 13:**
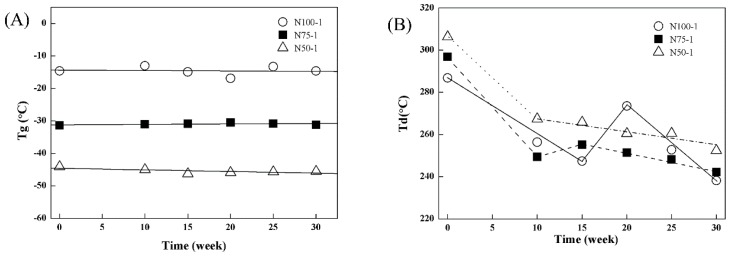
Change in thermal properties of the cross-linked P(TMC-*co*-CL) networks during *in vitro* hydrolytic degradation in PBS at 37 °C: (A) *T*g and (B) *T*d.

The *T*gs of the cross-linked P(TMC-*co*-CL) networks were not significantly changed (Figure 13A) while the Td decreased gradually (Figure 13B) under hydrolytic conditions *in vitro*, especially for the networks containing 50 mol % CL (N50-1), which had a decrease of 17.60% in *T*d after 30 weeks, it was due to more server destruction of the reticulate structure as indicated by the higher mass loss (Figure 9). Furthermore, the decrease in *M*_n_ and loss of CL content in composition of the sol fraction during the degradation process would also lower the value of *T*d, as reported in our previous works that had researched the degradation behavior of the linear P(TMC-*co*-CL) [42]. Obviously, there was a significant increase in Td of the cross-linked PTMC N100-1 after 20 weeks in PBS solutions (Figure 13B), which was caused by the first degradation of sol fractions. It resulted in the relative increase of gel contents and led to the increase of the thermal stability of the networks.

**Figure 14:**
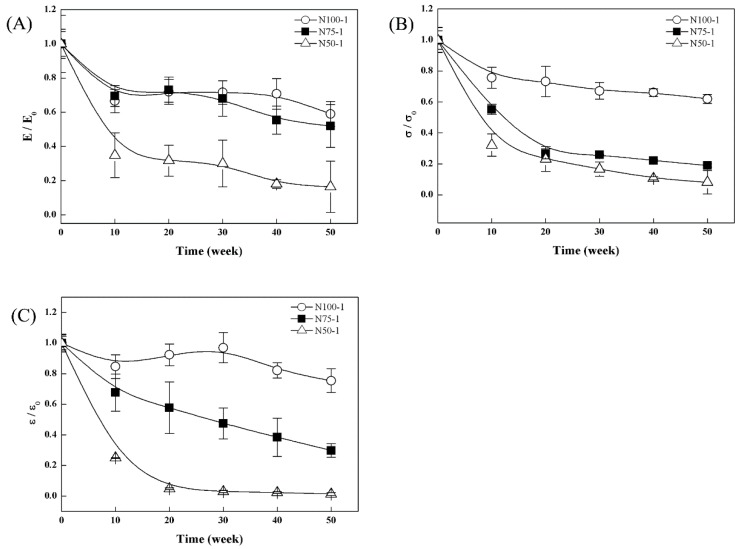
Change in physical properties of the cross-linked P(TMC-*co*-CL) networks during *in vitro* degradation in pH 7.4 PBS: (**A**) Young’s modulus (**B**) tensile stress and (**C**) tensile strain.

The changes in mechanical properties (Young’s modulus, *E*; tensile stress, σ; and tensile strain, ε) of the cross-linked P(TMC-*co*-CL) networks *in vitro* hydrolytic degradation were given in Figure 14. The results were normalized with respect to their initial values (*E*_0_, σ_0_ and ε_0_).

As seen in Figure 14, there was a clear tendency that all the Young’s modulus, tensile stress and tensile strain values of the cross-linked P(TMC-*co*-CL) networks decreased during the *in vitro* hydrolytic degradation. The addition of CL content in the copolymers resulted in significant decrease of mechanical properties of the cross-linked copolymer networks. For example, for the cross-linked copolymers, N50-1 contained 50 mol % CL, the modulus and tensile stress decreased to 16.4% and 8.1% of the initial values after 50 weeks, while the corresponding parameters of cross-linked PTMC N100-1 were 59.0% and 62.0% of the initial values. The tensile strain of the cross-linked polymers was shown in Figure 14C, and the trend was similar to that of modulus and tensile stress during the hydrolytic degradation.

The significant loss in the mechanical properties of the cross-linked copolymer networks was attributed to the higher mass loss caused by the instability of ester bonds in hydrolysis conditions. This result coincided with the hydrolytic degradation results already shown in Figure 9, indicating that the loss in mechanical properties of the cross-linked polymers was directly proportional to the mass loss.

### 3.4. In Vivo Degradation

The implants of all the cross-linked PTMC networks were subcutaneously implanted in the back of rats. The mass loss, thermal properties and mechanical properties of the implants as well as the histology at the sites of implantation were monitored and evaluated.

**Figure 15:**
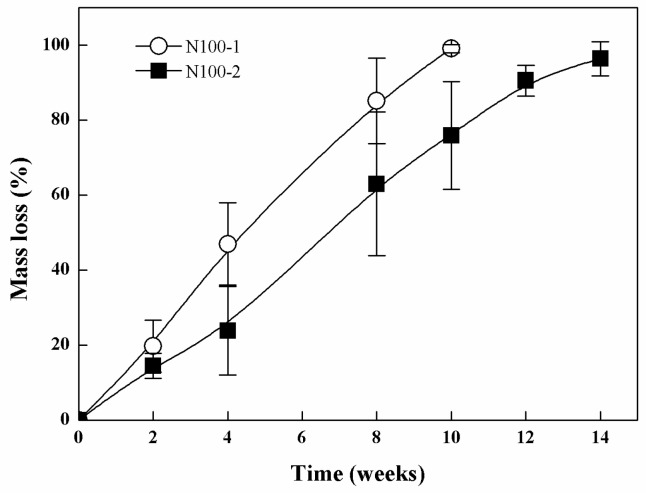
Mass loss of N100-1 and N100-2 at different implantation times in the back of rats.

The mass loss of the cross-linked PTMC networks *in vivo* was presented in Figure 15. The specimens of N100-1 were completely degraded after 10 weeks, while the N100-2 disappeared at week 14, indicating that the degradation rate decreased with increasing the cross-linker amount in the composition of the networks. It has been reported that high molecular weight PTMC degraded fast *in vivo* [24,25]; for instance, PTMC discs with molecular weight of 3.16 × 10^5^ g/mol subcutaneously implanted in the back of rats presented a weight loss of 96% in three weeks. Therefore, N100-1 and N100-2 exhibited a slower degradation rate as compared to the non-crosslinked PTMC mentioned above. The results indicated that cross-linking could lower and tailor the degradation rate of PTMC via the adjustment of cross-linking density.

**Figure 16:**
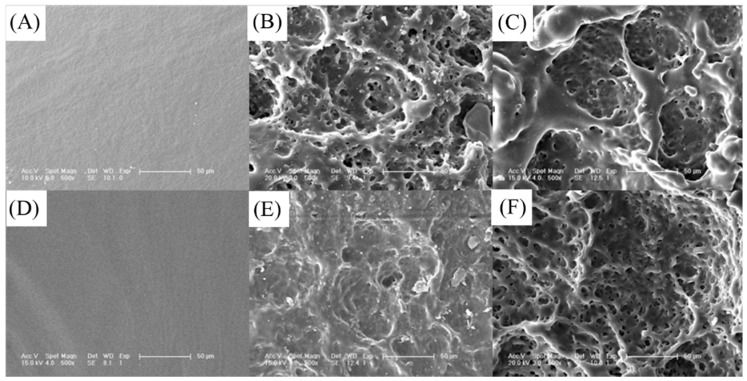
SEM micrographs of the surface of N100-1 (**A**–**C**) and N100-2 (**D**–**F**) after 0 (**A**,**D**); 2 (**B**,**E**) and 8 weeks (**C**,**F**) implanted in the back of rats. The scale bar was 50 μm.

The shape of the explants was not changed obviously, while the thickness decreased and the surface became rougher (The figures were not given), indicating that the degradation of the cross-linked PTMC *in vivo* proceeded via surface erosion mechanism. The surface morphology of the explants was observed by SEM as shown in Figure 16, and the surface was smooth without any cracking or pitting before implanting, while the surface of implants was eroded to a highly porous texture and numerous pits were visible after two weeks of implantation. With further degradation, the size and deepness of the pits were greater at eight weeks. Furthermore, the pit size observed on the surface of N100-2 was smaller *versus* that of N100-1 at each same time point. It further confirmed that the degradation rate of the cross-linked PTMC could be tailored by the cross-linking density: the higher the cross-linking density, the lower the degradation rater. The surface structure of cross-linked PTMC was very similar to that of PTMC films eroded *in vivo* [43,44]. It was likely that the degradation mechanism of these networks was surface enzymatic erosion. The rapid surface erosion of the cross-linked PTMC networks *in vivo* as well as being stable under hydrolytic conditions suggested that enzymes in body fluid played an important role in the degradation process. However, it is not clear yet which enzyme is dominant for the *in vivo* degradation of cross-lined PTMC. Meanwhile, body response, such as the accumulation of macrophages, may accelerate degradation [45].

**Figure 17:**
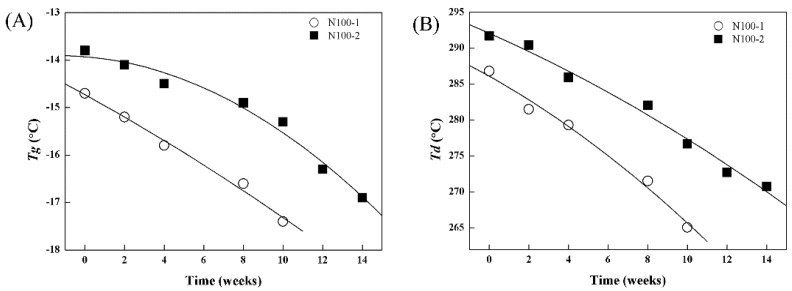
Change in thermal properties of N100-1 and N100-2 networks at different implantation times in the back of rats: (**A**) *T*g and (**B**) *T*d.

The thermal properties of the cross-linked PTMC degraded *in vivo* were presented in Figure 17. The thermal properties decreased as the degradation time increased, similar to that *in vitro* enzymatic degradation. It was quite evident that N100-2 had higher thermal stability than N100-1, which was attributed to the more stable structure caused by cross-linking when the amount of cross-linker increased.

**Figure 18:**
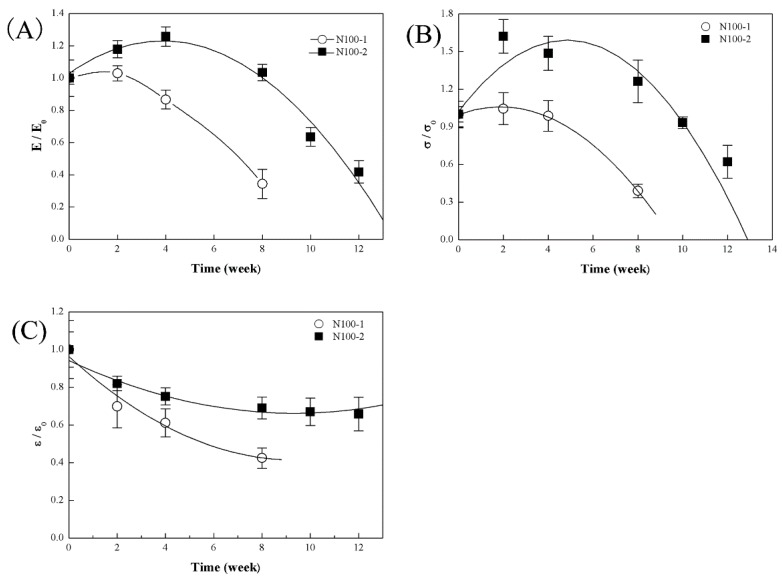
The changes in the mechanical properties of N100-1 and N100-2 networks during *in vivo* degradation: (**A**) Young’s modulus (**B**) tensile stress and (**C**) tensile strain.

Figure 18 shows the changes in mechanical properties of the cross-linked PTMC networks after implantation *in vivo*. Interestingly, the modulus of cross-linked PTMC N100-1 and N100-2 increased up to 103% and 118% of initial modulus in the first four weeks, and they then sharply decreased (Figure 18A). The tensile strength of cross-linked PTMC networks was shown in Figure 18B, and the trend was identical to that of modulus during implantation time. The increase in modulus and tensile strength was attributed to the anti-plasticization effect of hydrogen-bonded bridges established by water molecules penetrated in the inter-chain spaces of cross-linked polymers, once the explants were exposed to water for a short period, and the similar result was reported by other groups [26,46,47,48]. Different from the changes in modulus and tensile strength with degradation time, the networks underwent a marked decrease in tensile strain. N100-1 samples retained only 42.4% of their initial values, while N100-2 experienced a lower alteration of decrease to 65.8% of the initial value for 12 weeks *in vivo* degradation (Figure 18C). The samples of N100-1 were too weak to be clamped for tensile test and their mechanical properties could not be measured after degraded for 10 weeks. The same phenomena were found to N100-2 samples after 14 weeks degradation.

**Figure 19:**
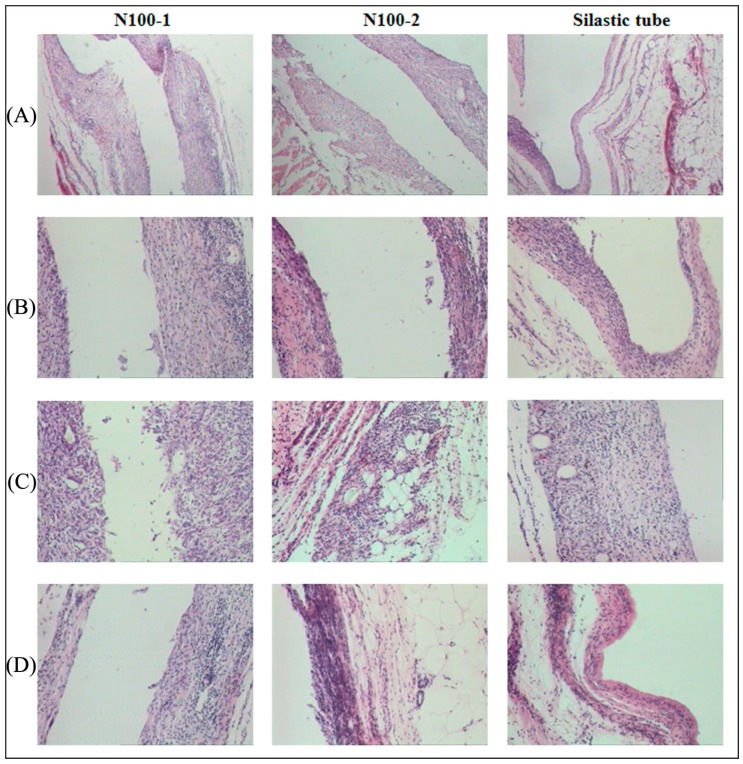
Histological sections of the tissue surrounding the implant at different stages of *in vivo* degradation: (**A**) 2 weeks at 40× magnification; (**B**) 2 weeks at 100× magnification; (**C**) 4 weeks at 100× magnification and (**D**) 12 weeks at 100× magnification.

To investigate the influence of degradation on the nature and extent of inflammatory and foreign body reactions, the cross-linked PTMC networks (N100-1 and N100-2) with a different cross-linker amount were implanted. As shown in Figure 19, at week 2, there was a minor inflammatory response at the site of implantation for both N100-1 and N100-2, the signs of formation of fibrous capsule and induction of vascularization were observed around the implants. Cells in the capsule were mainly fibroblasts and fibrocyte with a few multinucleated neutrophils and foreign-body giant cells, and the amount of cells was relatively higher than that around the silastic tube. However, no significant difference in the number of cells was found between the two samples.

The thickness of the capsule and the cellular content at the site of implantation increased slightly at week 4 for both elastomeric implants. After 12 weeks post implantation, these fibrous capsules composed of fibroblasts and fibrous tissue appeared to become thinner in a manner similar to other implanted biodegradable polymers [49,50,51], and the cellular content decreased for all the implants.

At the 4th week of implantation, some small fragments from the implanted networks were found on the tissue-implant interface, which was likely due to the erosion of the implants via phagocytosis, these polymeric fragments were absorbed and disappeared gradually with the prolonged degradation time, avoiding the removal of the degradation products from the site of implantation.

## 4. Conclusions

The degradation of cross-linked PTMC-based networks was investigated both *in vitro* and *in vivo*. The degradation rate of cross-linked PTMC networks was slower than that of the linear ones, indicating the enhanced resistance to degradation of PTMC via cross-linking with the adjustment of cross-linking density. With the comparison of an *in vitro* hydrolysis case, the faster degradation rate in lipase or *in vivo* was attributed to the essential role of enzymes in the erosion of cross-linked PTMC networks. Furthermore, surface morphology observation of the degradation samples demonstrated that the degradation was the surface erosion process. The cross-linked PTMC networks degraded extremely slowly in PBS, while the cross-linked P(TMC-*co*-CL) degraded dependent on the compositions. The higher the CL content, the faster the degradation. The cross-linked PTMC-based networks maintained better form-stability *versus* the non-crosslinked ones and were the promising candidates for potential clinical application in subcutaneous implants.

## Figures and Tables

**Table 1:** The characteristics of PTMC based networks ^a^.

No.	Monomers (mol)			Gel percentage ^b^ (%)	*M*n ^c^ (g/mol)	PDI ^c^	*T*g ^d^ (°C)	*T*d (°C)	*E* ^e^ (MPa)	σm ^f^ (MPa)	εm ^g^ (%)
	TMC	CL	BTB								
N100	100	0	0	n/a	274,700	1.07	−16.2	254.7	3.08 ± 0.18	3.32 ± 0.23	3570 ± 514
N100-1	100	0	0.01	19 ± 2	265,200	1.11	−14.7	286.8	3.18 ± 0.16	4.48 ± 0.59	2150 ± 212
N100-2	100	0	0.02	31 ± 2	288,400	1.06	−13.8	291.7	3.98 ± 0.20	6.69 ± 0.71	1350 ± 208
N75-1	75	25	0.01	11 ± 2	260,100	1.14	−31.4	296.8	2.69 ± 0.14	3.43 ± 0.12	3350 ± 173
N50-1	50	50	0.01	8 ± 3	240,500	1.21	−44.1	306.3	1.83 ± 0.23	1.30 ± 0.08	163 ± 10

^a^ Values are expressed as mean. Standard deviation (*n* = 3); ^b^ Values of gel fraction determined by using chloroform at room temperature; ^c^ Values of sol fraction determined by GPC at 35 °C using THF as the eluent; ^d^ measured from the second heating cycle; ^e^ *E* = elastic modulus; ^f^ σm = tensile stress; ^g^ εm = tensile strain.

## References

[1] Mehdikhani-Nahrkhalaji M., Fathi M.H., Mortazavi V., Mousavi S.B., Hashemi-Beni B., Razavi S.M. (2012). Novel nanocomposite coating for dental implant applications *in vitro* and *in vivo* evaluation. J. Mater. Sci. Mater. Med..

[2] Yang L.Q., Yang D., Guan Y.M., Li J.X., Li M. (2012). Random copolymers based on trimethylene carbonate and ε-caprolactone for implant applications: Synthesis and properties. J. Appl. Polym. Sci..

[3] Böstman O., Pihlajamäki H. (2000). Clinical biocompatibility of biodegradable orthopaedic implants for internal fixation: A review. Biomaterials.

[4] Weiler A., Hoffmann R.F., Stähelin A.C., Helling H.J., Südkamp N.P. (2000). Biodegradable implants in sports medicine: The biological base. Arthroscop.

[5] Witte F., Calliess T., Windhagen H. (2008). Degradable synthetische implantatmaterialien. Orthopade.

[6] Burkhart S.S. (2000). The evolution of clinical applications of biodegradable implants in arthroscopic surgery. Biomaterials.

[7] Allcock H.R., Chasin M., Langer R. (1990). Biodegradable Polymers as Drug Delivery Systems.

[8] Wang H., Dong J.H., Qiu K.Y., Gu Z.W. (1998). Synthesis of poly(1, 4-dioxan-2-one-*co*-trimethylene carbonate) for application in drug delivery systems. J. Polym. Sci. A Polym. Chem..

[9] Zhang Z., Foks M.A., Grijpma D.W., Feijen J. (2005). PTMC and MPEG-PTMC microparticles for hydrophilic drug delivery. J. Control. Release.

[10] Zhang Y., Zhuo R.X. (2005). Synthesis and drug release behavior of poly(trimethylene carbonate)-poly (ethylene glycol)-poly(trimethylene carbonate) nanoparticles. Biomaterials.

[11] Gu F., Younes H.M., El-Kadi A.O., Neufeld R.J., Amsden B.G. (2005). Sustained interferon-γ delivery from a photocrosslinked biodegradable elastomer. J. Control. Release.

[12] Yoshii T., Hafeman A.E., Nyman J.S., Esparza J.M., Shinomiya K., Spengler D.M. (2010). A sustained release of lovastatin from biodegradable, elastomeric polyurethane scaffolds for enhanced bone regeneration. Tissue Eng. A.

[13] Guan J., Stankus J.J., Wagner W.R. (2007). Biodegradable elastomeric scaffolds with basic fibroblast growth factor release. J. Control. Release.

[14] Pêgo A.P., Poot A.A., Grijpma D.W., Feijen J. (2001). Copolymers of trimethylene carbonate and ε-caprolactone for porous nerve guides: Synthesis and properties. J. Biomater. Sci. Polym. Ed..

[15] Schappacher M., Fabre T., Mingotaud A.F., Soum A. (2001). Study of a (trimethylenecarbonate-*co*-ε-caprolactone) polymer—Part 1: Preparation of a new nerve guide through controlled random copolymerization using rare earth catalysts. Biomaterials.

[16] Fabre T., Schappacher M., Bareille R., Dupuy B., Soum A., Bertrand-Barat J., Baquey C. (2001). Study of a (trimethylenecarbonate-c*o*-ε-caprolactone) polymer—Part 2: *in vitro* cytocompatibility analysis and *in vivo* ED1 cell response of a new nerve guide. Biomaterials.

[17] Song Y., Wennink J.W., Kamphuis M.M., Vermes I., Poot A.A., Feijen J., Grijpma D.W. (2010). Effective seeding of smooth muscle cells into tubular poly(trimethylene carbonate) scaffolds for vascular tissue engineering. J. Biomed. Mater. Res. A.

[18] Rocha D.N., Brites P., Fonseca C., Pêgo A.P. (2014). Poly(Trimethylene carbonate-*co*-ε-caprolactone) promotes axonal growth. PLoS ONE.

[19] Song Y., Wennink J.W., Kamphuis M.M., Sterk L.M., Vermes I., Poot A.A., Feijen J., Grijpma D.W. (2011). Dynamic culturing of smooth muscle cells in tubular poly(trimethylene carbonate) scaffolds for vascular tissue engineering. Tissue Eng. A.

[20] Papenburg B.J., Schüller-Ravoo S., Bolhuis-Versteeg L.A., Hartsuiker L., Grijpma D.W., Feijen J., Wessling M., Stamatialis D. (2009). Designing porosity and topography of poly(1, 3-trimethylene carbonate) scaffolds. Acta Biomater..

[21] Gui L., Zhao L., Spencer R.W., Burghouwt A., Taylor M.S., Shalaby S.W., Niklason L.E. (2011). Development of novel biodegradable polymer scaffolds for vascular tissue engineering. Tissue Eng. A.

[22] Jeong S.I., Kim B.S., Kang S.W., Kwon J.H., Lee Y.M., Kim S.H., Kim Y.H. (2004). *In vivo* biocompatibilty and degradation behavior of elastic poly(l-lactide-*co*-ε-caprolactone) scaffolds. Biomaterials.

[23] Zhang Z., Kuijer R., Bulstra S.K., Grijpma D.W., Feijen J. (2006). The *in vivo* and *in vitro* degradation behavior of poly(trimethylene carbonate). Biomaterials.

[24] Zhu K.J., Hendren R.W., Jensen K., Pitt C.G. (1991). Synthesis, properties, and biodegradation of poly(1,3-trimethylene carbonate). Macromolecules.

[25] Pêgo A.P., van Luyn M.J.A., Brouwer L.A., van Wachem P.B., Poot A.A., Grijpma D.W., Feijen J. (2003). *In vivo* behavior of poly (1, 3-trimethylene carbonate) and copolymers of 1, 3-trimethylene carbonate with d,l-lactide or ε-caprolactone: Degradation and tissue response. J. Biomed. Mater. Res..

[26] Pêgo A.P., Poot A.A., Grijpma D.W., Feijen J. (2002). *In vitro* degradation of trimethylene carbonate based (co)polymers. Macromol. Biosci..

[27] Albertsson A.C., Eklund M. (1995). Influence of molecular structure on the degradation mechanism of degradable polymers: *In vitro* degradation of poly(trimethylene carbonate), poly(trimethylene carbonate-*co*-caprolactone), and poly(adipic anhydride). J. Appl. Polym. Sci..

[28] Athanasiou K.A., Niederauer G.G., Agrawal C.M. (1996). Sterilization, toxicity, biocompatibility and clinical applications of polylactic acid/polyglycolic acid copolymers. Biomaterials.

[29] Sachlos E., Czernuszka J.T. (2003). Making tissue engineering scaffolds work. Review: The application of solid freeform fabrication technology to the production of tissue engineering scaffolds. Eur. Cell Mater..

[30] Karp J.M., Shoichet M.S., Davies J.E. (2003). Bone formation on two-dimensional poly (dl-lactide-*co*-glycolide) (PLGA) films and three-dimensional PLGA tissue engineering scaffolds *in vitro*. J. Biomed. Mater. Res. A.

[31] Engelberg I., Kohn J. (1991). Physico-mechanical properties of degradable polymers used in medical applications: a comparative study. Biomaterials.

[32] Pêgo A.P., Grijpma D.W., Feijen J. (2003). Enhanced mechanical properties of 1,3-trimethylene carbonate polymers and networks. Polymer.

[33] Bat E., Plantinga J.A., Harmsen M.C., van Luyn M.J., Zhang Z., Grijpma D.W., Feijen J. (2008). Trimethylene carbonate and ε-caprolactone based (co)polymer networks: mechanical properties and enzymatic degradation. Biomacromolecules.

[34] Bat E., Plantinga J.A., Harmsen M.C., van Luyn M.J., Feijen J., Grijpma D.W. (2010). *In vivo* behavior of trimethylene carbonate and ε-caprolactone-based (co)polymer networks: Degradation and tissue response. J. Biomed. Mater. Res. A.

[35] Timbart L., Tse M.Y., Pang S.C., Amsden B.G. (2010). Tissue response to, and degradation rate of, photocrosslinked trimethylene carbonate-based elastomers following intramuscular implantation. Materials.

[36] Jansen J., Boerakker M.J., Heuts J., Feijen J., Grijpma D.W. (2010). Rapid photo-crosslinking of fumaric acid monoethyl ester-functionalized poly(trimethylene carbonate) oligomers for drug delivery applications. J. Control. Release.

[37] Jansen J., Bosman M.B., Boerakker M.J., Feijen J., Grijpma D.W. (2010). Photo-crosslinked poly(trimethylene carbonate)-fumarate/*n*-vinyl pyrrolidone networks for the controlled release of proteins. J. Control. Release.

[38] Song Y., Kamphuis M.M., Zhang Z., Sterk L.M., Vermes I., Poot A.A., Feijen J., Grijpma D.W. (2010). Flexible and elastic porous poly(trimethylene carbonate) structures for use in vascular tissue engineering. Acta Biomater..

[39] Bat E., Kothman B.H., Higuera G.A., van Blitterswijk C.A., Feijen J., Grijpma D.W. (2010). Ultraviolet light crosslinking of poly(trimethylene carbonate) for elastomeric tissue engineering scaffolds. Biomaterials.

[40] Jansen J., Koopmans S.A., Los L.I., van der Worp R.J., Podt J.G., Hooymans J.M., Feijen J., Grijpma D.W. (2011). Intraocular degradation behavior of crosslinked and linear poly(trimethylene carbonate) and poly(d,l-lactic acid). Biomaterials.

[41] Yang L.Q., He B., Meng S., Zhang J.Z., Li M., Guan Y.M., Li J.X., Gu Z.W. (2013). Biodegradable cross-linked poly(trimethylene carbonate) networks for implant applications: Synthesis and properties. Polymer.

[42] Yang L., Li J., Meng S., Jin Y., Zhang J., Li M., Guo J., Gu Z. (2014). The *in vitro* and *in vivo* degradation behavior of poly(trimethylene carbonate-*co*-ε-caprolactone) implants. Polymer.

[43] Bat E., Plantinga J.A., Harmsen M.C., van Luyn M.J.A., Grijpma D.W., Feijen J. *In vivo* degradation of TMC and ε-CL(co)polymer networks. Proceedings of the 8th World Biomaterials Congress.

[44] Chapanian R., Tse M.Y., Pang S.C., Amsden B.G. (2009). The role of oxidation and enzymatic hydrolysis on the in vivo degradation of trimethylene carbonate based photocrosslinkable elastomers. Biomaterials.

[45] Tracy M.A., Ward K.L., Firouzabadian L., Wang Y., Dong N., Qian R., Zhang Y. (1999). Factors affecting the degradation rate of poly(lactide-*co*-glycolide) microspheres *in vivo* and *in vitro*. Biomaterials.

[46] Chapanian R., Tse M.Y., Pang S.C., Amsden B.G. (2010). Long term *in vivo* degradation and tissue response to photo-cross-linked elastomers prepared from star-shaped prepolymers of poly(ε-caprolactone-co-d,l-lactide). J. Biomed. Mater. Res. A.

[47] Karjalainen T., HiljanenVainio M., Malin M., Seppala J. (1996). Biodegradable lactone copolymers. III. Mechanical properties of ε-caprolactone and lactide copolymers after hydrolysis *in vitro*. J. Appl. Polym. Sci..

[48] Storey R.F., Warren S.C., Allison C.J., Puckett A.D. (1997). Methacrylate-endcapped poly(d,l-lactide-*co*-trimethylene carbonate) oligomers. Network formation by thermal free-radical curing. Polymer.

[49] Van Wachem P.B., van Luyn M.J., Olde Damink L.H., Dijkstra P.J., Feijen J., Nieuwenhuis P. (1994). Biocompatibility and tissue regenerating capacity of crosslinked dermal sheep collagen. J. Biomed. Mater. Res..

[50] Hooper K.A., Macon N.D., Kohn J. (1998). Comparative histological evaluation of new tyrosine-derived polymers and poly(l-lactic acid) as a function of polymer degradation. J. Biomed. Mater. Res..

[51] Holder W.D., Gruber H.E., Moore A.L., Culberson C.R., Anderson W., Burg K.J., Mooney D.J. (1998). Cellular ingrowth and thickness changes in poly-l-lactide and polyglycolide matrices implanted subcutaneously in the rat. J. Biomed. Mater. Res..

